# Lomi-Lomi

**Published:** 1871-01

**Authors:** 


### LOMI-LOMI.

### THE "MOVEMENT CURE" OF THE SOUTH SEA ISLANDERS.

The meaning of the Hawaiian or Sandwich Island word which stands at the head of this article is not translatable into any single expression in the English language. A description is required to do full justice.

The *lomi-lomi* (pronounced low-me-low-me), which as an institution is decidedly Polynesian, is a luxurious and healthful *passive motion* that is very much employed by the Hawaiians. It is used as a remedial measure in the treatment of disease, as a release from fatigue, and as a luxury and solace in hours of ease. It is a favor which travelers exchange with each other during the intervals of rest from the toilsome march, an act of kindness by which the wife soothes the fatigue of her husband after his daily labors, a service which the chief claims from his liegeman, and which the king receives as a prerogative from the hands of his subjects.

No one who has had experience as a Hawaiian traveler will fail to agree that the crowning act of generous hospitality of this most hospitable people to the well behaved traveler who sojourns in their midst, is the offer of the *lomi-lomi.*

There are many ways of administering the *lomi-lomi.* For our purpose, it will suffice to classify them under two divisions: First, the general, and second, the special. The special lomi-lomi is applied to any particular part affected, as to the head, in case of headache, to one of the limbs, or to any region of the body, in case of lameness or strain. The position of the recipient of this form may be either sitting or reclining, as is most convenient; but, when the lomi-lomi is general, the patient lies on a mat or on some low couch, convenient for the manipulator. Imagine yourself wearied and exhausted by a long journey, hungry, foot-sore and lame in every muscle, to such a degree that no position seems to give rest. Under ordinary circumstances, the probability is that, too weary for sleep, you would spend the night in rolling wretchedly, and rise in the morning unrefreshed and unfit to proceed on your journey.

Such would be the result in nine cases out of ten under the ordinary treatment of hospitality in civilized plains. But the Hawaiians have an appreciation of the physiological wants of the wearied system which it would be well for the people of other civilized and more enlightened countries to imitate. After water has been provided for washing and cooling purposes, or you have perhaps immersed yourself bodily into some clear, flowing stream, and have regaled yourself with a deliciously broiled fish and a sufficient number of finger dips into a calabash of *pori*—a favorite article of food made from a Polynesian esculent root, the *kaelo*—the lomi-lomi is proposed as a means of completing your refreshment and renovation. "What is this?" you ask. You are directed to lie at full length on a mat, and are immediately taken in hand by the artist, generally an elderly and experienced man or woman. The one who performs the lomi-lomi seizes your feet with strong hands, and commences manipulations which are neither kneading, nor squeezing, nor rubbing, but now like one and now like the other. Passing up to your body, the muscles of the calf, the thighs, the back and shoulders, each in turn receives appropriate treatment.

The degree of force used varies from the tenderest caress as to the severest grip. To those parts which are the most lame and sore, the feeling may at times be on the verge of painful, and, when at first applied to the legs of the weary novice, may cause him to shrink and complain, just as the first scalding dash of hot water terrifies the nerves of the inexperienced Turkish bather. But, by the time the *ha-huna-domi-lomi* (lomi-lomi doctor) has made one round, all distrust is gone, and you only regret that the delightful process must so soon come to an end. One skillful in the art of lomi-lomi comes to possess a kind of tact that enables him to graduate his touch and force to the wants of each case. At one moment his finger-points seem to dip down deeply and individualize each muscle, at another he skillfully presents only the rounded eminences of his palms, and soothes you with the magnetic influence of a gentle, undulatory motion. The skillful performance of lomi-lomi prevents the unpleasant effects of excessive exertion from being felt severely the next day—the stiffness, and lameness and soreness.

It also seems to impart a sufficient nervous power to enable one to sleep, so that the following morning you rise from your bed of clean Hawaiian mats with a feeling of freshness and rest that is quite surprising. Your muscles are supple, and your joints have an ease of motion that is unwonted. You are in fine, ready to commence another day's journey.

It is not uncommon for the natives to lie with their faces toward the ground, and have a child or youth of moderate weight walk over them from the heels to the nape of the neck, trampling them under foot, as grapes are trodden by the feet of the wine makers. This, although an inexplicable and alarming sight to the novice, we recommend as a most effective and salutary method of employing the lomi-lomi, especially appropriate when the lameness or weariness affects the back, the loins, or shoulders.—*Exchange.*

—A waggish journalist who is often merry over his personal plainness, tells this story of himself: I went to a chemist the other day for some morphine for a sick friend. The assistant objected to giving it to me without a prescription, evidently fearing that I intended to commit suicide. "Pshaw!" said I, "do I look like a man that would kill himself?" Gazing steadily at me a moment, he replied, "I don't know. It seems to me if I looked like you, I should be greatly tempted to get rid of myself."

